# Shisan C. Chen and his research on goldfish genetics

**DOI:** 10.1007/s13238-015-0236-3

**Published:** 2016-01-08

**Authors:** Lei Fu

**Affiliations:** Zhejiang Normal University, Jinhua, 321004 China

Shisan C. Chen (1894–1957) was an excellent geneticist (Fig. 1), who made outstanding contributions to research into the variation, evolution and heredity of goldfish and published many of his findings in scientific and educational journals. Shisan C. Chen’s research into goldfish remains highly regarded by the international scientific community and resulted in the illumination of this research field to scientists and the general public alike.

**Figure 1:**
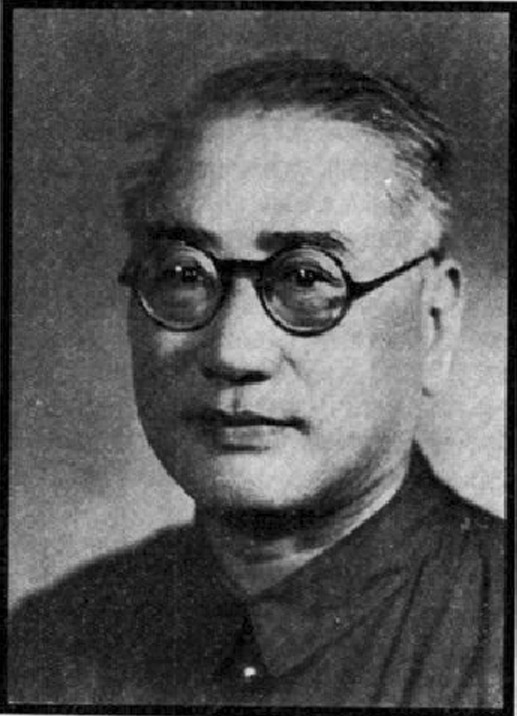
Shisan C. Chen (1894–1957)

Chen was born in Jiangsu Province in 1894. After graduating from China College in 1914, he was admitted to the University of Nanking, Jiangsu Province and majored in agriculture and forestry. In 1919 Chen passed an examination for overseas postgraduate studies held at Tsinghua University and subsequently carried out his postgraduate education in the United States at Cornell University and at the Department of Zoology in Columbia University, where he received his Master’s degree in 1921. Following an informative and enlightening period of research into genetics at Thomas Hunt Morgan’s laboratory, Chen returned to China in 1922. He was engaged as a professor by Southeastern University in Nanking and led research at the Biological Laboratory of the Science Society of China. In 1929 Chen was given the honor of the leadership of the Department of Biology at Tsinghua University and was among the first academicians of Academia Sinica in 1948 and of Chinese Academy of Sciences (CAS) in 1955. In 1953, he was appointed as a director of the zoology research unit of CAS.

As a geneticist, Chen carried out his research on goldfish and began his work in Nanking. He regarded goldfish as an appropriate study material as goldfish is native to China and can be easily cultivated from crucian based on their distinctive variations on the external characteristics. Moreover, the eggs and embryos of goldfishes provide convenient materials for experimental embryology as they can be fertilized *in vitro*. In his research, Chen undertook a comprehensive variety of genetic methodologies including cross breeding, embryology, cytology, statistical analysis and literature studies, etc., in order to gain a detailed understanding of goldfish genetics.

Chen studied variations on the external characteristics of goldfish, especially the shapes of fins and colors of skin (Chen, 1925). He investigated the inheritance of goldfish transparency scale by crossing various breeds of goldfish and mating domesticated breeds of goldfish and found a new characteristic called “transparent”, the inheritance of which was Mendelian. Furthermore, Chen proved the polypheny and incomplete dominant inheritance from this research and published his findings in *Genetics* in 1928 (Chen, 1928; Fig. 2). Moreover, in later experiments, Chen studied the inheritance of blue and brown colours in goldfish (Chen, 1934a).

**Figure 2:**
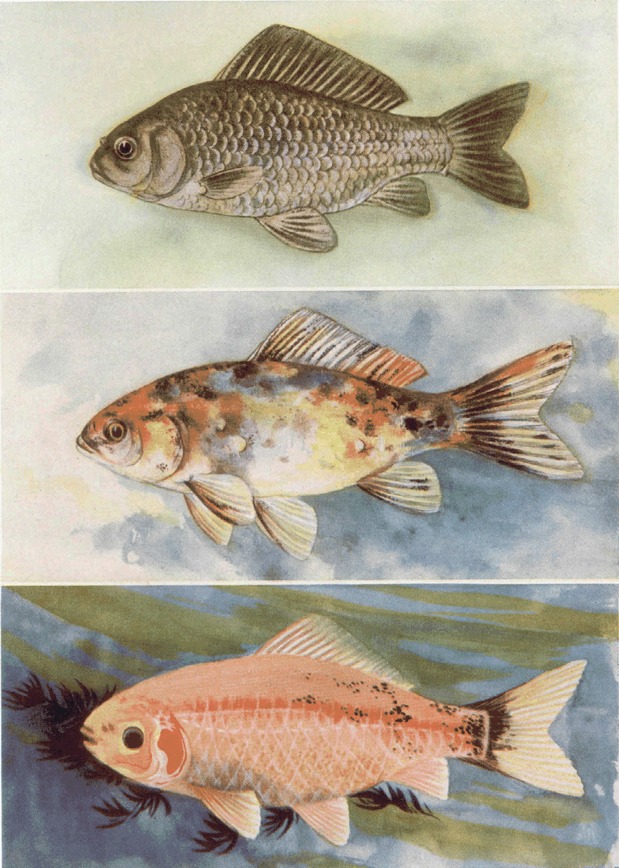
Goldfish by Shisan C. Chen (1928). From above: Normal scaled fish, Heterozygous mottled fish, Homozygous transparent fish

Chen also conducted research into the development of goldfish affected by being out of water, in distilled water and in solutions of alcohol and measured the specific gravity of various body-parts, organs and tissues of wild and domesticated goldfishes. These other research findings were published in *Contributions from the Biological Laboratory of the Science Society of China*, *Science*^1^, *China Journal*, *Biologia Generalis*, *Journal of Agriculture Science* and other prestigious journals and had a major impact on Mendelian genetics (Chen, 1959). He also published his research findings in *Journal of Tsinghua University*. Thus, his students in Tsinghua University gave him the nickname: A Boss of Goldfish Store (Chen, 1934b).

By collating and researching a large number of historical documented observations of goldfish breeding in ancient China, Chen was able to propose that goldfish had originated from crucian and that their domestication began in the Southern Song Dynasty. Moreover, Chen found that directional breeding wasn’t practiced until Qing Dynasty and pot raising played a key role in the course (Chen, 1959). These findings have contributed to our understanding of the variations and inheritance of goldfish and have provided an insight into the history of biological selection processes in ancient China.

Chen placed a great importance on biology education in secondary and elementary school. He delivered a speech on goldfish in *New Education Review* in 1926, in which he suggested that goldfish could be used in biology courses to increase students’ interest in biology, and to demonstrate the morphology, physiology, embryo and development, variation and inheritance of animals as experimental materials (Chen, 1926). On his return from America, Chen found there were few textbooks concerning fundamental principles of biology and therefore resolved to write a book *General Biology* for college students in 1924, which became widely adopted in China. In 1933, he revised the textbook for high school students with a new name *Fuxing High School Textbook Biology* and published it by Commercial Press, in which he supplemented some advances of biology and replaced some materials with his own research, especially the inheritance of goldfish transparency scale (Chen, 1933). This textbook was very popular and adopted till 1951.

Shisan C. Chen won international acclaim for his work on goldfish genetics and created a model for integrating scientific research and scientific historiography research with the dissemination of science.

## Footnotes

1 This magazine was launched by the Science Society of China in 1915.
